# Genes of the RNASE5 pathway contain SNP associated with milk production traits in dairy cattle

**DOI:** 10.1186/1297-9686-45-25

**Published:** 2013-07-16

**Authors:** Lesley-Ann Raven, Benjamin G Cocks, Jennie E Pryce, Jeremy J Cottrell, Ben J Hayes

**Affiliations:** 1 Biosciences Research Division, Department of Primary Industries Victoria, 5 Ring Rd, Bundoora 3086, Australia; 2 La Trobe University, Bundoora, VIC 3086, Australia; 3 Dairy Futures Co-operative Research Centre, Bundoora, VIC 3086, Australia

## Abstract

**Background:**

Identification of the processes and mutations responsible for the large genetic variation in milk production among dairy cattle has proved challenging. One approach is to identify a biological process potentially involved in milk production and to determine the genetic influence of all the genes included in the process or pathway. Angiogenin encoded by *angiogenin, ribonuclease, RNase A family 5* (*RNASE5*) is relatively abundant in milk, and has been shown to regulate protein synthesis and act as a growth factor in epithelial cells *in vitro.* However, little is known about the role of angiogenin in the mammary gland or if the polymorphisms present in the bovine *RNASE5* gene are associated with lactation and milk production traits in dairy cattle. Given the high economic value of increased protein in milk, we have tested the hypothesis that *RNASE5* or genes in the RNASE5 pathway are associated with milk production traits. First, we constructed a “RNASE5 pathway” based on upstream and downstream interacting genes reported in the literature. We then tested SNP in close proximity to the genes of this pathway for association with milk production traits in a large dairy cattle dataset.

**Results:**

The constructed RNASE5 pathway consisted of 11 genes. Association analysis between SNP in 1 Mb regions surrounding these genes and milk production traits revealed that more SNP than expected by chance were associated with milk protein percent (P < 0.05 significance). There was no significant association with other traits such as milk fat content or fertility.

**Conclusions:**

These results support a role for the RNASE5 pathway in milk production, specifically milk protein percent, and indicate that polymorphisms in or near these genes explain a proportion of the variation for this trait. This method provides a novel way of understanding the underlying biology of lactation with implications for milk production and can be applied to any pathway or gene set to test whether they are responsible for the variation of complex traits.

## Background

Bovine milk is a valuable and widely consumed source of high-value protein and fat. The cytological mechanism of milk synthesis and secretion in cattle is well-described [1], however, the molecular mechanisms responsible for variations in the components of milk require further investigation given the importance of these processes for human nutrition. In dairy cattle, the heritability of milk protein yield has been estimated to be approximately 23% [2]. If the polymorphisms contributing to milk traits were identified, this information could be used in breeding programs to increase milk protein yields. Furthermore, identification of the gene pathways involved will contribute to the understanding of the mechanisms that regulate lactation and to the development of new approaches to improve milk production and the value of milk proteins for human nutrition.

Genome-wide association studies (GWAS) have become a popular methodology to identify genomic regions containing variants affecting complex traits. In dairy cattle, GWAS have identified several regions of the genome associated with variation in milk protein content [3-7]. This method requires stringent thresholds to avoid high rates of false positives caused by multiple testing. As a result, it does not perform well for traits that involve many genes of small effect and cannot identify relationships between genes adequately. An alternative approach is to use prior biological knowledge to select gene pathways that are likely to be involved in milk production, and thus to limit association analyses to SNP that are within or in close proximity of the genes in these pathways. While this approach reduces the number of polymorphisms that can be identified, it has the advantage of using lower significance thresholds since the whole genome is not tested and multiple testing is greatly reduced, leading to more power to detect associations of smaller effect [8]. This approach can also be used to test whether a particular gene pathway is associated with a quantitative trait such as protein yield in milk.

Genetic variations in milk protein genes including the four caseins and the major whey proteins ß-lactoglobulin and a-lactalbumin have been studied in detail, with documented polymorphisms affecting fat and protein concentrations [9]. However, these polymorphisms only account for a fraction of the genetic variation of traits like protein yield. Minor milk proteins may also be important regulators of these traits since they can have a functional role in milk or the mammary gland. One interesting candidate gene for the regulation of lactation is the gene *angiogenin, ribonuclease, RNase A family 5* (*RNASE5*). The protein RNASE5 is a member of the ribonuclease A superfamily, i.e. proteins known for their potent action as RNA cleaving enzymes. However, RNASE5 is an ancestral member of the RNase A family that has unique and distinct functions from other members of this family [10]. RNASE5 is well known for its role in blood vessel formation and has been linked to cancer pathogenesis [11,12]. It is also involved in the regulation of ribosomal RNA synthesis, protein synthesis in prostate epithelial cells and has a growth factor function in epithelial and endothelial cell types [13,14]. While expressed in many different tissue types, secretory epithelial cells appear to be the primary location for RNASE5 synthesis [15]. RNASE5 has been detected in bovine milk at a concentration ranging from 4 to 19 μg/mL, but its role in milk remains unclear [15,16] and may include an antimicrobial function [15,17]. In the bovine genome, the gene *RNASE5* is duplicated but no functional role explains this duplication [17]. The presence of RNASE5 in milk and hence mammary tissue and the fact that it has a regulatory role in protein synthesis in epithelial cells suggest that it could have a function during lactation. Knowledge on the mechanism of RNASE5 synthesis and action may contribute to identify a source of genetic variation associated with milk production traits and establish a regulatory role for RNASE5 in the mammary gland.

Variation in the expression of *RNASE5* could result not only from polymorphism in the gene itself, or its cis-acting regulatory elements, but also from upstream regulators or downstream mechanisms. The mechanism of action of the RNASE5 protein involves several key processes. RNASE5 binds to the endothelial cell surface via a 42 kDa α-actin and this complex activates cell-associated protease and subsequent degradation of the basement membrane [18]. RNASE5 also binds to a 170 kDa cell surface receptor, a complex that sets off signal transduction cascades, and undergoes translocation to the nucleus via endocytosis [19] where it enhances rRNA transcription [20] and rRNA processing [14]. The ribonucleolytic activity of RNASE5 is required for its biological function [20]. RNASE5 exhibits cell density-dependence and cell surface receptor activity, which suggests that it acts in a paracrine way [19]. There is also evidence that RNASE5 is expressed in mammary epithelial cells [21].

This study explores the role of the RNASE5 pathway in milk production traits. We hypothesised that RNASE5 may be associated with different aspects of lactation in the mammary gland, and therefore that the *RNASE5* gene and the genes involved in the RNASE5 pathway could contain polymorphisms affecting milk and protein production in dairy cattle. To test this hypothesis, first we reviewed the literature for genes involved in the RNASE5 pathway, including direct upstream and downstream regulators. Next, we used a linear regression model to test the association between a set of SNP located 500 kb on either side of each of the genes in the RNASE5 pathway and milk production traits (at a P ≤ 0.05 experiment-wise threshold).

## Methods

### Construction of the RNASE5 pathway

In this study, an association analysis between SNP in the genes of the RNASE5 pathway and several milk production and reproductive traits in dairy cattle was performed. Current research aimed at investigating the role of RNASE5 in epithelial cells and in the pathogenesis of amyotrophic lateral sclerosis (ALS) and cancer has provided insights into its cellular role. Based on these studies, we identified proteins known to regulate or interact with RNASE5 and constructed a pathway gene set. This isolated subset of identified genes was hypothesised to be collectively specific to the cellular role of RNASE5. We limited the gene pathway to genes for which the proteins showed evidence of a direct binding interaction with RNASE5, a clear role in the already-characterised mechanism of action of RNASE5 or those that were significantly up-regulated by RNASE5. We excluded genes such as *AKT* involved in broad-acting cellular processes e.g. cell signalling and growth factors. While these proteins are functionally important, we wanted to avoid any interference from similar molecular pathways and processes, which may be involved in milk and protein production independently of the RNASE5 pathway.

The genomic location of the genes in the RNASE5 pathway was determined using the Bovine Genome Build 4.0 in the NCBI database [22]. To identify the bovine ortholog of the human *small nucleolar RNA* gene (*SNORD15A*), one of the genes in the pathway, we conducted a sequence comparison using the basic alignment search tool (BLAST) version 2.2.25 in the NCBI database [23]. We identified the start and stop positions of each gene and expanded the region located 500kb on either side of the gene to reflect the extent of linkage disequilibrium (LD) in Holstein cattle [24], and to ensure that there was a sufficient number of SNP in the windows analysed (on the Bovine SNP50 BeadChip [25]) and thus that there was a reasonable chance that at least one SNP would be in moderate to high LD with a causal mutation, given the highly variable nature of pair-wise LD. These collective SNP provided the pathway gene SNP set for analysis. After quality control performed according to [8], the total 50k SNP chip data set consisted of 43 115 SNP. Of these, 211 SNP were within our gene windows (see results for more detail). Two thousand one hundred and fifty-four Holstein-Friesian bulls were genotyped for the 50k chip and at each SNP, genotypes were 0, 1 or 2, based on the number of copies of the first allele (first alphabetically in top format). The phenotypes for the bulls were the daughter trait deviations (essentially the average of the bulls’ daughters records for these traits, corrected for fixed effects such as herd, year and season) for milk yield, fat yield, fat percent, protein yield, protein percent and fertility, as previously described [26,27]. To account for population structure, a relationship matrix derived from a pedigree dating back to the 1940’s was fitted in the model, as described below.

### SNP association analysis

An association analysis was used to determine the relationship between SNP polymorphisms within the RNASE5 pathway and milk production and reproductive traits. Statistical analyses were performed using ASReml [28]. The linear mixed model used for the SNP analysis, for each SNP in turn, was

$$y=1_{n}\mu +X\beta +\mathrm{Zu}+e$$

where **y** is a (number of bulls × 1) vector of daughter trait deviations, **1**_n_ is a vector of 1s, μ is the overall mean, **X** is the vector of bull genotypes (0,1 or 2), β is the effect of the second allele of the SNP, **Z** is a design matrix allocating daughter trait deviations to bulls, **u** is a vector of polygenic effects and **e** is the vector of random residuals. The polygenic breeding values were fitted as random effects following a normal distribution $N\left(0,A\sigma_{a}^{2}\right)$ where **A** is the expected relationship among individuals constructed from the pedigree and $\sigma_{a}^{2}$ is the polygenic genetic variance.

Significant SNP were selected at a p-value threshold of P ≤ 0.05. The proportion of significant SNP in the pathway was then the number of significant SNP divided by the number of SNP tested. To determine an appropriate experiment-wise significance threshold, a random permutation method was used. This approach used only SNP in gene regions. A list of 23 022 uniquely annotated bovine genes were selected from the Ensembl-Biomart database [29]. Then, we chose 11 genes (the same number as in our pathway) at random and the genomic regions spanning 500 kb on either side of these genes were used to construct the SNP sets for analysis. This procedure was replicated 10 000 times. The SNP were analysed in ASReml [28] using the mixed linear model described above and the resulting null distribution was expressed as the proportion of significant SNP. This method controls for potential bias in the proportion of significant SNP by testing SNP in gene regions versus intergenic regions. The ratios of significant SNP from the permutations were compared to the observed distribution to determine experiment-wise significance for each trait.

We calculated the false discovery rate (FDR) at P ≤ 0.05 with the observed data, and the permutated data as [4]

$$m\cdot \frac{P}{S}$$

where, *m* is the number of tests, P is the probability value of the F-test for the linear regression and S is the proportion of significant SNP at P ≤ 0.05. We also analysed the correlations between significant SNP for each trait to determine the extent of pleiotropy.

This study considered genomic regions spanning 500 kb on either side of each gene in the RNASE5 pathway. These genomic regions were chosen to reflect the extent of linkage disequilibrium in Holstein-Friesian cattle; however, other genes that lie within these regions may produce conflicting results. For example, a gene harbouring a mutation with a well characterised effect on fat percentage, *diacylglycerol O-acyltransferase 1* (*DGAT1*) was within the window for *plectin*, a gene in the RNASE5 pathway [30]. To control the effect of this gene in our analysis, we repeated the association study and permutations with phenotypes corrected for the effect of *DGAT1* (in fact, the SNP most highly associated with the polymorphism in this gene). Each animal was assigned a new phenotype for which the effect of the *DGAT1* SNP from the experimental GWAS was subtracted as a multiple of the number of common alleles present.

## Results

### Construction of the RNASE5 pathway

We identified a pathway that includes the genes known to specifically interact with RNASE5 i.e. genes coding for proteins that interacted with RNASE5, that had a clear role in the already characterised mechanism of action of RNASE5 or those up-regulated *in vitro* by RNASE5. We avoided cell signalling and growth genes which have broad functions within the cell to avoid overlap with irrelevant cell functions. RNASE5 appears to act as a highly regulated switch. Studies have identified synthetic and non-synthetic RNASE5 binding proteins in an attempt to regulate angiogenesis as a cancer treatment. However, many of these proteins are not naturally occurring or are unlikely to function during normal RNASE5-mediated processes such as angiogenesis, so the corresponding coding genes were not included in the analysis.

**Figure 1:**
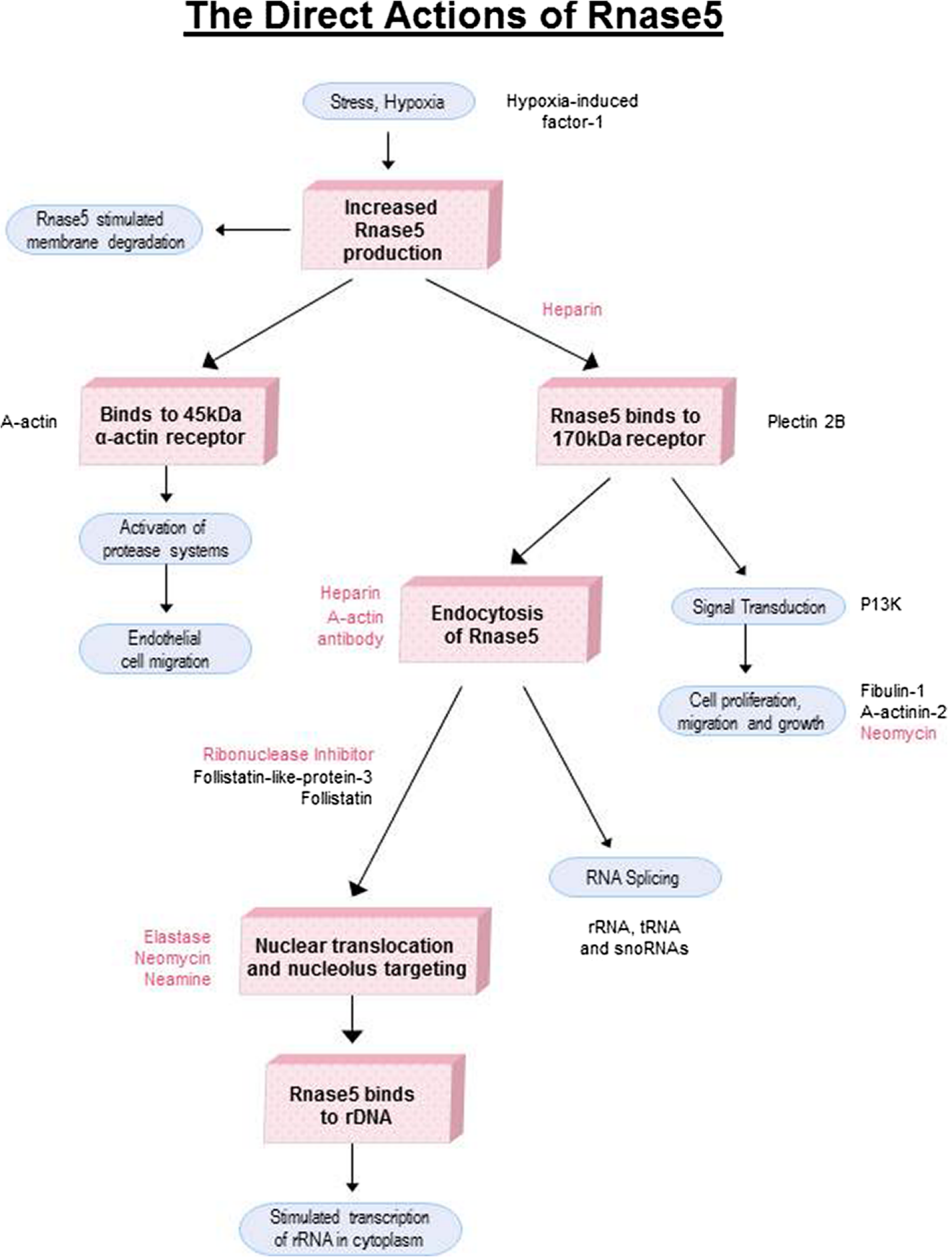
**The direct pathway of action of *RNASE5. *** The proposed mechanism of action as determined from a review of the literature. Hypoxic stress triggers transcription of *RNASE5*[37]. *RNASE5* triggers the expression of two cell-density dependent receptors. At higher cell densities, *RNASE5* binds with the endothelial cell surface via a 42-kDa α-actin receptor which stimulates basement membrane degradation [18]. This complex triggers plasminogen activation which in turn stimulates endothelial cell migration and angiogenesis [18,45]. Once the cell density decreases, the 170 kDa *RNASE5* receptor is synthesized [19]. Binding interactions with this 170 kDa cell surface receptor trigger endocytosis of *RNASE5*[19]. Once internalised, *RNASE5* triggers a series of cell signalling pathways including second messenger responses, MAPK activation and phosphorylation of Erk1/2 which stimulate cell proliferation, migration and growth [46-48]. From the cytoplasm, *RNASE5* is translocated to the nucleus where it enhances rRNA transcription [20]. *RNASE5* cleaves rRNA and tRNA and in turn recombinant angiogenin has been shown to act as a cytotoxic tRNase that abolishes protein synthesis [49-51]. We also revealed a significant number of activators and inhibitors of this pathway e.g. NO, however, only proteins were considered [52]. The path of the *RNASE5* protein is highlighted in red boxes, with blue boxes representing the subsequent processes. The genes involved in this process are dictated alongside each process with activators and inhibitors noted outside each process in red (inhibitors) and black (up-regulators and other known binding proteins).

Information was taken from a large number of publications describing the primary actions of RNASE5 as well as interactions with other proteins (e.g. [31] and references therein). We identified over 100 proteins among which 11 met our criteria for analysis (Figure 1; Table 1). The genes, *RNASE5* and its homologue *RNASE4*, were the first obvious candidates. RNASE5 increases protein synthesis in murine epithelial cells and shows differential expression in lactating mammary gland [21,32,33] (e.g. accession numbers: E-GEOD-5258, E-GEOD-29119, E-MTAB-37, E-MTAB-62, E-GEOD-20081, E-TABM-420, E-GEOD-3952, E-TABM-683, E-TABM-683, E-GEOD-20081, E-GEOD-3526, E-MTAB-62, E-GEOD-7307). These genes are transcribed from the same promoters and are functionally homologous [16,34]. *RNASE5* also shows homology to the *LOC783225 angiogenin-1-like* gene (83.6% DNA identity) and *angiogenin 2* (75.8% DNA identity). Since, these genes lie in close proximity to *RNASE5*, only the *RNASE5* gene region was considered in order to avoid multiple testing. The *RNASE5* promoter contains two binding sites for the stress response gene *hypoxia-inducible factor-1* (*HIF1A*) [35]. In an oxygen deprived environment, HIF1A increases the extracellular concentration of RNASE5, binds growth factors and enhances *RNASE5* mRNA expression [36,37]. Three cell surface receptor genes were identified: *α-actin 2* (*ACTA2*), *α-actinin 2* (*ACTN2*) and *plectin 2B* (*PLEC*). ACTA2 is a well-known RNASE5-binding protein involved in cell proliferation, migration and invasion [38,39]. The plectin-2B receptor is known to function as the RNASE5 cell receptor (GF Hu, personal communication). RNASE5 also interacts with several stimulatory proteins i.e. fibulin (FBLN1), follistatin (FST) and follistatin-like protein 3 (FSLP-3). RNASE5 directly binds to fibulin *in vivo*[40] and contains follistatin binding domains [41]. Follistatin-like-3 is functionally and structurally homologous to follistatin but has not been shown to interact directly with RNASE5 [42]. However, because of its homology to follistatin and its characteristic nuclear localisation, it is a candidate gene for the RNASE5 pathway [42]. Three other genes were identified from a previous analysis on *RNASE5* in murine epithelial cells. Microarray data indicate that *SNORD15A* (*Small nucleolar RNA, C/D box 15A*) is strongly down-regulated by *RNASE5/FST* in mouse mesodermal cells [32,33] (M McDonagh, personal communication). In mouse, it has been shown that *SNORD15A* is involved in the maturation and post-transcriptional modifications of tRNA, mRNA and rRNA but the bovine ortholog has not been isolated. BLAST analysis [23] showed that the human *SNORD15A* gene shared 89% similarity with the bovine *Regulatory Factor X* gene (*RFX7*), which was thus included in the RNASE5 pathway to represent *SNORD15A*. Ribosomal protein L22-like 1 (RPL22L1) functions as a component of the 60S ribosomal subunit and is localized to the nucleus via binding interactions with rRNA [43]. Both, *RPL22L1* and *SP140* (sp140 nuclear body protein) genes were significantly up-regulated in a microarray analysis of murine epithelial cells treated with RNASE5 [32,33] (M McDonagh, personal communication). These genes are also differentially expressed in murine and human mammary gland (accession numbers: E-GEOD-3526, E-GEOD-20081, E-MTAB-62, E-MTAB-37, E-AFMX-4 [44]).

**Table 1:** RNASE5 pathway gene set and characteristics of the different SNP regions

**Gene**	**Chr**	**SNP region**		**Nb SNP**	**Number of SNP significant**					
		**Start**	**Stop**		**Protein yield**	**Protein %**	**Fertility**	**Fat yield**	**Fat %**	**Milk yield**
**ACTA2**	26	10578865	11592811	26	7		5	6	2	6
**ACTN2**	28	7393500	8441168	16	2			1	2	2
**FBLN-1**	5	122206254	123286509	22	4	10	5			3
**FSLP-3**	7	41906484	42911309	20	3	9	1	2		
**FST**	20	26797134	27802564	13	2	8		1	4	1
**HIF1**	10	75457880	76502552	17	1	1	2			3
**PLEC2**	14	190799	1212951	16	16	15	3	15	16	16
**RFX7**	10	54530392	55670964	25	2	2			1	
**RNASE4/5**	10	25298775	26315348	23	5			1		4
**RPL22L1**	1	98326431	99330809	17	4	5	2	1		1
**SP140**	2	121890759	122935785	16	2	7			2	4
**Total nb SNP**				**211**	**48**	**57**	**18**	**27**	**27**	**40**

### SNP within the RNASE5 pathway are significant for some milk production traits

Genomic regions affecting milk production and reproductive traits were identified by association analysis using the RNASE5 pathway gene set constructed above. Using the Illumina Bovine SNP50 BeadChip, we identified a set of 211 SNP within a 1 Mb region surrounding each gene in the pathway [25]. Linear regression analysis with ASReml [28] revealed significant associations between the SNP within the RNASE5 pathway gene set and some milk production and reproductive traits in livestock, at P < 0.05. Many SNP were associated with protein yield, protein percent and milk yield while fewer SNP were associated with fertility, fat yield and fat percent (Table 1). For each trait tested, at least half of the genes in the pathway contained significant SNP (P < 0.05). The genes *PLEC*, *ACTA2*, *FBLN1*, *SP140*, *FST* and *FSLP3* contained a large number of significant SNP for protein percent and protein yield. In fact, for protein yield there was at least one significant SNP for each gene in the RNASE5 pathway. Eight genes were associated with SNP significant for protein percent.

**Figure 2:**
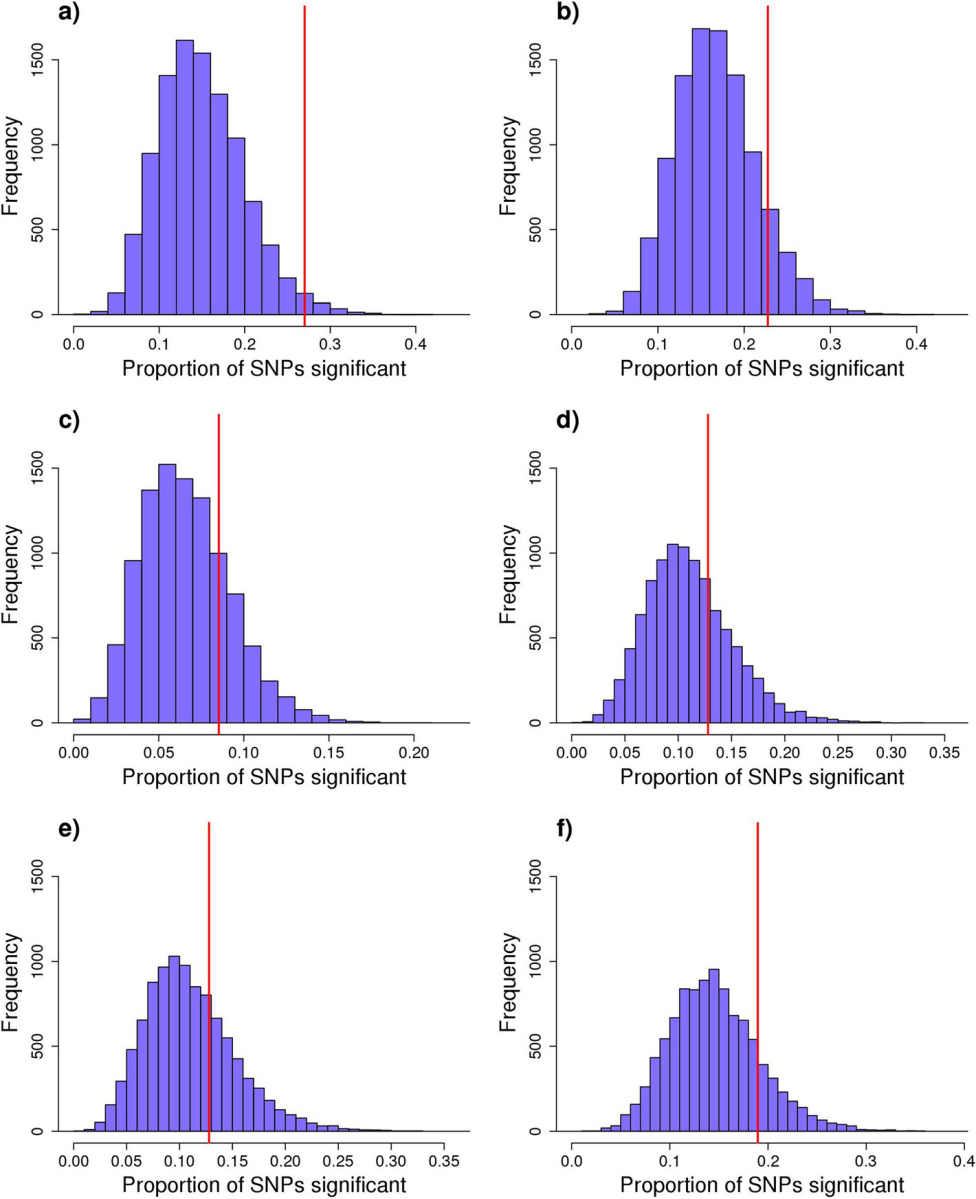
**Permutation tests for protein, milk and fertility related traits using 50 k SNP data.** Blue represents the null hypothesis distribution. SNP sets were randomised from 11 gene regions with 10 000 repeats and expressed as a ratio (P < 0.05). The red line represents the experimental ratio for each trait of interest: **a)** protein percent (f-value = 0.270), **b)** protein yield (f-value = 0.227), **c)** fertility (f-value = 0.085), **d)** fat yield (f-value = 0.128), **e)** fat percent (f-value = 0.128) and **f)** milk yield (f-value = 0.190).

Permutation testing was used to determine the experiment-wise significance of each pathway tested (e.g. accounting for the multiple testing of 211 SNP). For protein percent, only 1.8% of SNP sets from the 10 000 permutations exceeded the observed ratio of significant to non-significant SNP (Figure 2). This means that for protein percent the experiment-wise P-value is less than 0.018. For protein yield, 10.9% of the SNP sets within the permuted distribution exceeded the observed SNP ratio of 0.227 (Figure 2). No other traits approached experiment-wise significance and FDR was high compared to previous studies (Table 2). We investigated the extent of pleiotropy for polymorphisms in the RNASE5 pathway by calculating the correlation between SNP effects for significant SNP (Table 3). SNP effects for milk volume were negatively correlated with SNP effects for protein percent, fat percent and fat yield (r = −0.720, -0.915 and −0.549, respectively) but were highly correlated with protein yield (r = 0.902). SNP effects for fat percent were highly correlated with SNP effects for fat yield and protein percent (r = 0.838 and 0.763). Interestingly, most of the aforementioned correlations showed higher correlations than the whole genome dataset (Table 3). This could be consistent with the fact that these targeted groups of SNP are more highly associated with the traits than would be expected from a random group of SNP. However, the difference between the two sets of correlations was not significant based on a paired *t* test.

**Table 2:** False discovery rates (%)

**Trait**	**Current study**	**Pryce*et al.*(2010)**
Protein yield	21	17
Protein %	18	8
Milk yield	26	13
Fat yield	39	18
Fat %	39	13
Fertility	586	53

**Table 3:** Correlations between SNP for milk production and reproductive traits within the RNASE5 pathway (N = 211) and the whole genome (N = 43115)

**RNASE5 pathway**						
	**Protein %**	**Protein yield**	**Fertility**	**Fat percent**	**Fat yield**	**Milk yield**
**Protein %**	1.000					
**Protein yield**	−0.352	1.000				
**Fertility**	−0.056	0.215	1.000			
**Fat %**	**0.763**	**−0.761**	−0.018	1.000		
**Fat yield**	***0.615***	−0.356	0.212	**0.838**	1.000	
**Milk yield**	***−0.720***	**0.902**	0.184	**−0.915**	*−0.549*	1.000
**Whole genome**						
	**Protein %**	**Protein yield**	**Fertility**	**Fat percent**	**Fat yield**	**Milk yield**
**Protein %**	1.000					
**Protein yield**	0.126	1.000				
**Fertility**	−0.116	0.244	1.000			
**Fat %**	*0.571*	−0.419	−0.121	1.000		
**Fat yield**	0.195	*0.538*	0.210	0.397	1.000	
**Milk yield**	−0.421	**0.845**	0.286	*−0.692*	0.383	1.000

Correlations were constructed from the solutions of linear regressions; strong correlations are indicated in bold characters and moderate correlations in italic characters; standard errors for each analysis are ±0.069 for the Rnase 5 pathway and ±0.004 for the whole genome. Fertility is measured as calving interval.

On bovine chromosome 14, *PLEC* and *DGAT1* lie in close proximity, and *DGAT1* is known to contain a polymorphism affecting milk volume and particularly fat percent in dairy cattle [30,53]. To determine if the *DGAT1* polymorphism was responsible for the significant result in our analysis, we re-ran the association model for protein percent and protein yield, with the effect of the polymorphism in *DGAT1* removed from the phenotypes (e.g., phenotypes corrected for *DGAT1* genotype effect). Removal of the effect of the polymorphism in *DGAT1* did not alter the overall significance of either the SNP in the *PLEC* gene or the RNASE5 pathway.

## Discussion

In this study, we identified an RNASE5 pathway as a potentially novel pathway associated with milk protein percentage in dairy cattle. We described a gene set that includes the genes coding for the main proteins interacting with RNASE5 and most likely to mediate RNASE5 action in mammary epithelial cells. SNP within or in close proximity to these genes explain some of the variation in milk production traits in dairy cattle, particularly for protein percent. RNASE5 may play a role in regulating lactation in the mammary gland similar to its mechanism in angiogenesis, i.e. it may be associated with the proliferation of mammary secretory cells, leading to higher volumes of milk, and higher yields of protein and fat. Certainly, RNASE5 is expressed in the mammary tissue of lactating mice, and is down-regulated during involution of the mammary gland. Since, RNASE5 is known to have a role in protein synthesis it may contribute to increased protein synthesis in milk, however other functions such as regulation of stress responses and apoptosis and even angiogenesis may also contribute to the observed regulatory role in milk. In epithelial cells, RNASE5 is internalized and localizes to the nucleus to stimulate protein synthesis and it is involved in increasing levels of rRNA and their processing. Thus, a similar mechanism may occur in mammary secretory cells but this hypothesis requires further testing.

A potential limitation of our study is the density of the SNP panel, which combined with the extended low level of linkage disequilibrium in dairy cattle populations makes the localisation of genetic variants to small genomic regions difficult e.g., [26]. This limited the confidence in the assignment of the associations observed between significant SNP and nearby genes in the RNASE5 pathway. We attempted to deal with this in two ways. First, we compared the significance of our results to those obtained from a very large number of random permutations, where SNP were chosen to be within the same distance (500 kb) from a randomly chosen set of 11 genes. Protein percent in particular was still significant at the experiment-wise level set by permutation testing. Second, we attempted to account for genes known to harbour genetic variants affecting milk production traits. Specifically, we re-ran the analysis on phenotypes corrected for the effect of the *DGAT1* mutation. However, we still acknowledge the limitation of the marginal SNP density in our experiment. The experiment could be repeated in the future with whole-genome sequence data, for example.

The 11 genes identified in the RNASE5 pathway include mainly genes involved in cell binding, structure and motility or with regulatory roles. Interestingly, most of the genes functioning in the extracellular space were not associated with any significant SNP. These include *RNASE5*, *ACTA2* and *ACTN2*. *HIF1* was associated with only one significant SNP located within the gene. This may suggest that regulation of protein percent by RNASE5 occurs primarily via signalling mechanisms within the cell. Analysis of the sequence variation within these genes may elucidate the true causal variants. In addition, the degree of pleiotropy identified from our correlation analysis suggests that the RNASE5 pathway potentially affects several production traits, and at a higher level of pleiotropy than a similarly sized set of randomly chosen SNP.

The pathway-based approach used here may be more powerful to identify associated SNP than a GWAS methodology relying on the large number of associations detected by whole-genome studies. On the one hand, since the focus of this method is to exploit previous information, it may miss new information but on the other hand, it provides a way to analyse large datasets. For example, low frequency variants explaining less of the variation can potentially be identified (due to lower significance thresholds). However, we acknowledge that this method is less relevant when the associated gene pathways are poorly described in the literature. Some of the biological processes in which the *RNASE5* gene and the RNASE5 pathway are involved have been identified but many remain uncharacterised. These include associations between alternative RNASE5 induced pathways, temporal aspects, cell receptor expression and triggers. Additionally, it is likely that there are other binding partners that vary with cell type and environmental conditions.

## Conclusions

The link between the genes in the RNASE5 pathway and variation in protein percent in bovine milk suggests that RNASE5 may have a hitherto undescribed biological mechanism in the mammary gland.

The gene set method that we applied here to the RNASE5 pathway could be used to rapidly assess the role of other emerging pathways and functions with a genetic validation relevant *in vivo*. Pathways with well-established biological steps or those with new biological knowledge as it becomes available are well suited to this type of analysis. The method can potentially identify associations of smaller effect than in standard GWAS, given the less stringent significance threshold required as a result of reduced multiple testing.

## Competing interests

The authors declare that they have no competing interests.

## Authors’ contributions

LG performed the GWAS and bioinformatic analysis in this study and drafted the manuscript. BC and BH conceived the study and participated in the design and co-ordination of this study. JP designed the GWAS and linear regression protocol and assisted the data analysis. JC described the content and function of RNASE5 in milk. All authors read and approved the manuscript.
